# Understanding the Relation Between Sleep and Health in Adulthood: Daily Experiences to Long-Term Health Outcomes

**DOI:** 10.1093/geroni/igaa057.2173

**Published:** 2020-12-16

**Authors:** Hye Won Chai, David Almeida, Soomi Lee

## Abstract

An increasing number of studies evinces the significant role of sleep in health outcomes including physical symptoms, cardiometabolic functioning, and chronic health conditions. To further advance the field’s knowledge on sleep and health in adulthood, it is necessary to have an integrative understanding of this topic that pulls together short-term determinants to long-term health consequences of sleep. As such, this symposium places diverse aspects of sleep across multiple contexts, ranging from predictors and consequences of sleep in daily life to the role of sleep in long-term changes in health across adulthood. The first paper by Lee and colleagues examines the role of daily positive and negative events as precursors of concurrent and next-day sleep duration. The second paper by Joo and colleagues addresses the moderating role of nightly sleep duration in the association between daily stressor severity and intensity of headaches. The third paper by Chai and colleagues explores how the association between daily emotional well-being and cardiometabolic syndrome differs by sleep deficiency and by age. The fourth paper by Sin and colleagues focuses on daily affective vulnerability to short sleep duration as a risk factor for developing chronic conditions 10 years later. The final paper by Reither and colleagues assesses within-person changes and between-person differences in restorative sleep and their associations with body mass trajectories across 15 years. The discussant, Dr. Soomi Lee, will integrate key points from the studies, discuss the utilization of diverse measurements of sleep, and address considerations for future research on sleep and health.

